# Crossmodal correspondence of elevation/pitch and size/pitch is driven by real-world features

**DOI:** 10.3758/s13414-024-02975-7

**Published:** 2024-10-26

**Authors:** John McEwan, Ada Kritikos, Mick Zeljko

**Affiliations:** School of Psychology, The University of Queensland, St. Lucia, QLD 4072 Australia

**Keywords:** Multisensory integration, Neural mechanisms, Scene perception

## Abstract

Crossmodal correspondences are consistent associations between sensory features from different modalities, with some theories suggesting they may either reflect environmental correlations or stem from innate neural structures. This study investigates this question by examining whether retinotopic or representational features of stimuli induce crossmodal congruency effects. Participants completed an auditory pitch discrimination task paired with visual stimuli varying in their sensory (retinotopic) or representational (scene integrated) nature, for both the elevation/pitch and size/pitch correspondences. Results show that only representational visual stimuli produced crossmodal congruency effects on pitch discrimination. These results support an environmental statistics hypothesis, suggesting crossmodal correspondences rely on real-world features rather than on sensory representations.

## Introduction

Crossmodal correspondences (CMCs) can be loosely defined as a tendency to associate sensory features from different sensory modalities in a consistent way. For example, the visual feature of size is associated with the auditory feature of pitch such that large size is associated with low pitch, and small size is associated with high pitch (Gallace & Spence, 2006). When these features align in a stimulus, the features are termed ‘congruent’, while a mismatch is termed ‘incongruent’. The congruency of these features produces a behavioural influence on a variety of tasks such as detections, discrimination, ambiguity resolution, implicit association, and multisensory binding (Ben-Artzi & Marks, 1995; Bien et al., 2012; Marks, 1987; Parise & Spence, 2008; Zeljko et al., 2019; Zeljko et al., 2021). Other examples of associations between simple, or low-level, visual and auditory features include elevation/pitch, lightness/pitch, brightness/loudness, shape/pitch and motion/pitch (Ben-Artzi & Marks, 1995; Bernstein & Edelstein, 1971; Clark & Brownell, 1976; Gallace & Spence, 2006; Marks, 1987; Melara, 1989). There are also examples of crossmodal correspondence where the feature is complex, or high-level. Multiple studies have observed associations between musical genres and colours where faster music was associated with lighter, more saturated colours, and slower music was associated with darker, desaturated colours, for instance (Barbiere et al., 2007; Palmer et al., 2013; Spence & Di Stefano, 2022; Wells, 1980). There has been much work into the origins of these associations, which has coalesced into four main ideas. It is important to clarify that these proposed origins are not exclusive or necessarily exhaustive. The current consensus is that it is highly likely that different CMCs have different origins, and that some CMCs are contributed to by multiple factors (Fernandez-Prieto et al., 2017; Motoki et al., 2023; Spence, 2011).

The most influential of these ideas is the *environmental statistics hypothesis*, which proposes that associated sensory features usually co-occur in the environment, and that the sensory system internalizes these co-occurrences, responding preferentially when they re-occur, and sub-optimally when they do not (Parise et al., 2014; Spence, 2019). Supporting this is evidence that there is a crossmodal pair between both visual size and pitch, and haptic size and pitch that follow the same size/pitch mapping (large size/low pitch, small size/high pitch) (Hamilton-Fletcher et al., 2018). First, this suggests that the association is not related to the specific modality it is encoded from but is related to the real-world size which is experienced by both vision and touch. Second, the specific mappings reflect the real-world physical relationship between object size and resonant frequency, whereby larger objects vibrate at lower frequencies, creating lower frequency sound waves, which are then perceived as being lower in pitch. Similar evidence of environmental correlations has been observed in the elevation/pitch crossmodal correspondence. By recording sounds using a directional microphone, researchers have demonstrated correlations between pitch and elevation in both natural and artificial environments whereby a higher pitch predicts a higher spatial location of origin for the sound, most evidently within a frequency band of 1–6 kHz (Parise, 2016; Parise et al., 2014). Other evidence supporting the environmental statistics hypothesis includes artificial induction of CMC-like pairings through training paradigms (Ernst, 2007; Fifer et al., 2013). Although the success of these studies in inducing an artificial CMC-like association does not offer direct support for the other CMCs under investigation being environmentally driven, it provides evidence for the viability of a learned multisensory association between features.

The *structural hypothesis* suggests that CMCs are a result of unintended interactions produced by the neural encoding of these features in the brain (Spence, 2011, 2020b; Walker et al., 2010). Most variations of this argument propose that CMC associations are not necessarily related to environmental correlations but are instead caused by either coincidental compatibility among stimuli encoding across modalities, or optimization of neural coding in the brain (Ramachandran & Hubbard, 2001; Smith & Sera, 1992; Walsh, 2003). The only exception to this arbitrariness is the specific proposal that CMCs may arise from hardwired neural interactions, which have evolved in response to environmental correlations. Parise (2016) describes this idea in brief, but many variations of the structural hypothesis continue to propose a mechanism which is unrelated to the environment. The strongest evidence for the structural hypothesis is rooted in studies of human infants expressing crossmodal correspondences. Walker et al. (2018) tested for the presence of elevation/pitch crossmodal correspondences in newborn infants (median age: 44 h), finding that they showed a significant preference in looking behaviour for congruent pairings of the audiovisual stimuli. Similar results have been found by Mondloch and Maurer (2004) in older (2-year-old) infant samples and Walker et al. (2010) in 3- to 4-month-old infants. Walker et al. argue that the presence of CMCs at such an early age constitutes evidence against claims that these associations are learned, because the infants would not have had sufficient exposure to the environmental correlations in the limited period immediately after birth. Discussing the possibility of prenatal learning of an elevation/pitch association, the authors suggest that it is unlikely due to the limited visual experience and the lack of a consistent spatial orientation in the womb. Alongside studies of infants, there is also some theoretical support for a structural hypothesis in the literature of neural activation. For example, both brightness and loudness stimulation are characterized by physiological excitation which increases proportionally with the stimulus magnitude (Stevens, 1957). Some authors have proposed that this shared system of neural coding creates a compatibility effect between the crossmodal information (Spence, 2011). Other proposed mechanisms include a potential parietal brain region which encodes for stimulus magnitude amodally (Smith & Sera, 1992; Walsh, 2003), or proximity of the brain regions which encode for each crossmodal feature (Ramachandran & Hubbard, 2001). However, there are difficulties for the structural hypothesis in explaining why CMCs are relative rather than absolute (Brunetti et al., 2018; Walker & Walker, 2016).

Third, the *semantic hypothesis* refers specifically to CMC congruencies which may be driven by a match in terms of their identity or meaning (Spence, 2011; Walker et al., 2012). For example, in English, the spatial feature of elevation is described using the terms ‘high’ and ‘low’. Similarly, the auditory feature of pitch is described using the terms ‘high’ and ‘low’. The semantic hypothesis proposes that the elevation/pitch correspondence (and others) are a result of the shared linguistic terms used for both features. The evidence cited for this is typically the observation that the feature pairings do correspond to the shared terms (e.g., high elevation/high pitch are congruent). Gallace and Spence (2006) induced size/pitch congruency effects while substituting the high/low pitches for the spoken words ‘high’ and ‘low’, which they interpreted to be a linguistic mediation of the size/pitch correspondence, showing that this concept can be expanded beyond direct sharing of adjectives. Evidence in non-English languages is mixed. Catalan, spoken in north-eastern Spain, does not share descriptive adjectives for elevation and pitch. Speakers of Catalan show an elevation/pitch congruency in the expected manner, arguing against semantic matching (Fernandez-Prieto et al., 2017). However, the effect was greater in those participants who spoke English as a second language, suggesting that linguistics may still mediate the effect. There is also strong evidence of CMC effects in animals including dogs (Korzeniowska et al., 2019, 2022), chimpanzees (Ludwig et al., 2011), and tortoises (Loconsole et al., 2023), as well as in human infants (Mondloch & Maurer, 2004; Ozturk et al., 2013; Walker et al., 2010). The range of non-linguistic subjects who exhibit these effects constitutes fair evidence that the semantic hypothesis, while relevant, cannot fully explain crossmodal correspondence.

Finally, the *affective hypothesis* is more abstract than the other three and suggests CMCs are driven by a matched affective response in the observer (Spence, 2020a). Researchers began to consider this theory as CMC research began to deal with associations which cannot be explained in terms of the other three hypotheses. Music and colour for instance (Levitan et al. 2015; Palmer et al., 2013) were shown to be associated by matching colour or music with emotion, then music and colour. Musical pieces are consistently matched to colours where the music and colour both shared an emotional association. Taste and shape are another example where traditional explanations cannot fully explain how these two modalities are consistently matched (Turoman et al., 2018). Turoman et al. (2018) showed that pleasantness and threat were consistently mediating the association between roundness/sweetness, angularity/sourness, and angularity/bitterness. Finally, odour-colour pairings have been partially explained by emotional mediation (Schifferstein & Tanudjaja, 2004). Darker colours are typically perceived as smelling more intense than lighter colours, and this effect is mediated by the mean difference in pleasure ratings between the crossmodal features. These affective CMCs can range from innately affective stimuli such as tastes and smells to complex stimuli where the affective response is learned, such as music, and so encompass a broad range of pairings.

Part of the difficulty in studying crossmodal correspondences, and the variety of hypothesized origins for their existence, is the wide range of pairings which exist (see Spence, 2011, for a review). This makes it unlikely that they all share the same underlying cause, and it is plausible that different CMCs have different underlying mechanisms while producing similar behavioural results. Our current study restricts itself to considering simple CMCs, that is, CMCs where the stimulus properties are basic and low-level in nature (such as lightness, visual brightness, pitch, size). These simple CMCs exist in contrast to what we would term complex CMCs, where the feature is a mixture of integrated simple features, and likely involves high-level processes (art, music, complex flavours such as wine). With regard to the discussed hypotheses, the affective hypothesis can be excluded from consideration as the simple features of elevation, size, and pitch do not typically produce affective responses.

Something that has not yet been considered in the literature is that humans do not have direct access to veridical features as they exist in the environment. The veridical elevation or size of an object is not inherently known, it is constructed from a variety of sensory information. One part of that sensory information is the elevation or size of the stimulus retinotopically, but this information does not necessarily predict veridical elevation or size. For instance, tilting one’s head to 90 degrees to the side would change an object’s retinotopic elevation, but not its veridical position in space. Similarly, objects can be brought closer to the eye, changing their retinotopic size but not their veridical size. We can apply this logic to all features in the environment. There can always be a disconnect between our sensory experience of the stimulus, and the nature of the veridical world, as evidenced by the many sensory illusions developed over the years. Despite this, people are able to maintain their perception of the veridical feature through a capacity referred to as perceptual constancy. Perceptual constancy refers to the capacity to maintain a constant percept of a feature, even when the sensations it produces change (Sperandio & Chouinard, 2015; Walsh & Kulikowski, 1998). In the above examples of elevation and size, perceptual constancy maintains our perception of the feature, even when the retinotopic sensation it produces changes, by incorporating other sources of information such as distance or orientation. From this integration of information, we can form a mental representation of the feature, distinct from the retinotopic information. As a result of this distinction, the retinotopic feature and the representational feature are not just different reference frames of the same concept such as elevation, they are entirely different neural mechanisms. Retinotopic elevation does not necessarily share any commonalities of elevation as a mental representation.

**Fig. 1:**
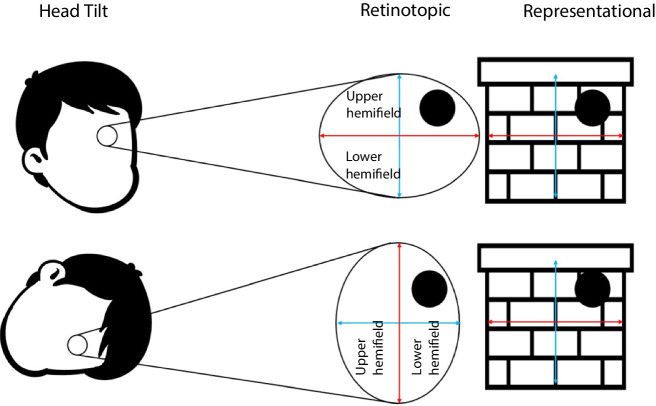
Diagram illustrating the difference between retinotopic and representational features. In both cases, the representational feature is consistent in the environment, while the retinotopic feature varies with head tilt. The blue line represents an up/down axis, and the red line represents a left/right axis

Considering this divide, we will use the terms ‘retinotopic’ and ‘representational’ throughout the paper to refer to this distinction between immediately available sensory features as they exist on the eye, and the conscious experience of veridical features constructed from integrated sensory information. Retinotopic will refer to a stimulus feature as it exists on the retina, which is reflected in the early cortical visual system, while representational will refer to the stimulus feature as it is represented by the brain after integration into the scene, often occurring later in the sensory stream. When we refer to manipulating representational size, we attempt to alter the subject’s mental representation of the feature, as if this was a veridical three-dimensional (3D) scene, through the use of monocular depth cues. Figure 1 depicts this distinction.

In relation to crossmodal correspondence, we believe this distinction between retinotopic features and the representational features is relevant to the question of CMC origins. If we treat our sensory experience as a distinct type of feature from our representation of the feature, we can explore this distinction with greater nuance. Regarding the environmental hypothesis, we can posit that only the representational feature of elevation or size would produce crossmodal correspondence effects. In these features, it is the veridical feature in the environment which produces its associated crossmodal stimulus, not our retinotopic experience of the feature. This argument should be treated with caution, however, because it not universal. Some features, such as visual depth, produce luminance information which can be reliably extracted on a retinal level (Hibbard et al., 2023; Samonds et al., 2012). In the case of CMC pairings which utilize features that produce reliable statistical regularities in a two-dimensional (2D) image, it is possible that the retinotopic experience may predict an environmental correlation and induce crossmodal correspondence. We believe that in the case of elevation and size this is not the case, and that they should only respond to representational variation. Parise et al. (2014) showed that higher pitched sounds are more likely to come from above, demonstrating the viability of the environmental hypothesis. Tilting one’s head 90 degrees to the right as described earlier would not change the position of these sounds, merely the retinotopic hemifield which encodes the visual stimulus. Higher pitches would now appear to come from the left of a viewer’s retinotopic field but remain above them in space. In a more applied example, gaze is often directed to examine the ground, making retinotopic elevation a measure of distance rather than real-world elevation. Turning this example to size/pitch, imagine a series of identical steel drums lined up diagonally extending away from the observer. From a retinotopic perspective, these drums will become smaller as they stretch into the distance, but in a viewer’s mental representation of size, they will remain the same size. This capacity to maintain a mental representation of size despite sensory changes is referred to as *size constancy* (Sperandio & Chouinard, 2015). If each drum were struck, each would resonate at the same frequency. Thus, only the representational quality of size is correlated with pitch. If size/pitch reflects an internalized association drawn from correlations of stimuli in the environment, that association should be sensitive to changes in representational size but not retinotopic size.

Despite the importance of this distinction between retinotopic and representational features for the environmental hypothesis, it remains unclear from the current literature which type of feature is driving crossmodal correspondence. Leaving aside Parise et al. (2014), which utilizes unisensory rather than multisensory pitch localizations, the only study to our knowledge that has examined this question previously is Carnevale and Harris (2016). They showed that pitch-biased perceptions of up/down visual motion correspond along elevation/pitch lines, and the direction of elevation biasing was determined by both a body-centric and gravity-centric reference frame. While this might suggest that we should expect to find an effect in both retinotopic and representational features, there are two important caveats. First, Carnevale and Harris (2016) tilted their participants to achieve a disconnect between body-centric and gravity-centric mapping. Tilting introduces vestibular and proprioceptive feedback cues regarding orientation in space, and may have influenced results (Diener & Dichgans, 1988; Smith & Reynolds, 2017). Our study takes steps to avoid the use of tilt in comparing retinotopic and representational features. The second difference is that retinotopic features and body-centric features are not quite the same thing, and so our retinotopic versus representational dichotomy is orthogonal to their body versus gravity dichotomy. Retinotopic features are much more closely related to early neural signals, while body-centric perception is still very much a high-level construct (Longo et al., 2010). Other than this study, much of the evidence for one or the other is circumstantial and not a focus of the original experiments. Many publications treat retinotopic and representational features as the same concept, referring to their features only as ‘elevation’ or ‘lightness’, without consideration of what exactly is meant by these terms. It is possible that this stems from the relative robustness of CMC effects compared with some other multisensory effects. CMCs are typically regarded as bidirectional and relative, and likely represent a mix of high and low-level processing, meaning they can be observed even without rigorous control over experimental stimuli (Motoki et al., 2023; Spence, 2011, 2019; Spence & Deroy, 2013). The relative nature of CMCs could be taken as implicit support for a representational or scene-integrated feature account of elevation/pitch and size/pitch, because both these ideas appeal to the involvement of higher-level cognitive processes in modulating the congruency effect (Brunetti et al., 2018; Chiou & Rich, 2012; Gallace & Spence, 2006). It does not necessarily support such an account, however, and so further research is required. The purpose of the current experiment was to resolve this ambiguity regarding the nature of the irrelevant CMC feature by proposing a direct test of the feature driving crossmodal correspondence, and by extension, an indirect test of the environmental statistics hypothesis in two common CMCs, elevation/pitch and size/pitch.

To consider the differences between retinotopic and representational features in crossmodal correspondence, we conduct two experiments, each consisting of two blocks. Experiment 1 investigates elevation/pitch. In the representational block of Experiment 1, the visual feature of elevation will be held retinotopically constant but be allowed to vary representationally by reorienting participants towards the top or bottom of the screen, then presenting the target at fixation. This will allow us to test for the presence of a CMC without variation in the retinotopic position. In the retinotopic block of Experiment 1, we will do the reverse. The visual feature of elevation will be held representationally constant but vary in position retinotopically by reorienting participants gaze to either the top or bottom of the screen, then presenting the target centrally. In all experiments, participants make an auditory discrimination of a pitch (high or low) in dynamic noise. The auditory and visual features will be presented concurrently to induce CMC effects. The critical variation between the representational and retinotopic blocks is the nature of the visual feature paired with the pitch. Experiment 2 investigates size/pitch. In the representational block of Experiment 2, we hold the visual feature of size retinotopically constant, in a 3D depth illusion. By placing the visual feature at different distances in the depth illusion while keeping retinotopic size constant, we can examine the size/pitch correspondence to representational size change, without retinotopic differences through size constancy. Finally, in the retinotopic block of Experiment 2, we hold the visual feature constant in representational size. In the depth illusion, this means the visual feature will be smaller when it is further away, maintaining its representational size but creating a difference in retinotopic size (Sperandio & Chouinard, 2015). We will measure accuracy in discriminating the tone, with a significant improvement to accuracy in the congruent pairings over incongruent pairings being indicative of a congruency effect.

In accordance with the environmental hypothesis, we predict that crossmodal congruency effects will be sensitive to the representational feature of the CMC pairing, and not the retinotopic feature. In both Experiment 1 and Experiment 2, congruent pairing of the respective CMC will produce significantly greater discrimination accuracy than the incongruent pairing in the representational block. Conversely, the retinotopic block will show no significant differences in discrimination accuracy between the congruent and incongruent pairing in both Experiment 1 and Experiment 2. This will suggest that low-level crossmodal correspondence effects depend on the mental representation of a real-world change rather than merely a change in immediate retinotopic experience, supporting the hypothesis that CMC effects are drawn from the natural environment.

## Method

### Participants

Twenty-seven participants (seven male, 19 female, one non-binary; age: 20.2 years ± 3.29) were recruited for Experiment 1 and 26 participants (eight male, 19 female; age: 25 years ± 10.39) were recruited for Experiment 2. Participants were recruited through a mix of The University of Queensland research participation system for first year students and The University of Queensland paid research participation system. Participants were compensated either course credit (in the case of first-year students) or $20 (in the case of paid participants) for their time. Sample size was determined based on effect sizes in previous CMC work on discriminations (Zeljko et al., 2019). For an effect size of 0.25 (Cohen’s F), and a power of 0.8, 24 participants were required for both experiments. The experiment was approved by The University of Queensland’s School of Psychology ethical review process.

### Design

All experiments followed a 2 (visual feature) × 2 (pitch) × 2 (feature type) within-groups design and consisted of 128 trials. Participants made unspeeded discrimination responses on the pitch of an audiovisual stimulus. The visual component of this audiovisual stimulus consisted of binary variation in the visual component of each relevant CMC. These were elevation (high or low in visual space) for Experiment 1, or size (big or small) for Experiment 2. The auditory component to be discriminated was a pure tone (high or low in pitch) embedded in dynamic noise. The feature type condition consisted of the representational condition, where the visual feature was representational in nature, and the retinotopic condition, where the visual feature was retinotopic in nature. The feature type conditions were blocked and completed in a counterbalanced order. During each feature type block, the irrelevant visual feature type was held constant, for example, the retinotopic size/pitch block would hold representational size constant for all trials. Before beginning each feature type block, participants completed a 16-trial practice block to familiarize themselves with the task, and verbally confirmed their understanding of the task with the experimenter. In analysis, the visual feature and pitch variables were collapsed along CMC congruency lines to form the composite IV of congruency. The following combinations were considered ‘congruent’: high elevation/high pitch and low elevation/low pitch and small/high pitch and large/low pitch. The reverse pairings were categorized as incongruent.

### Materials

All experiments were completed on either Dell Optiplex 9030 AIO or Dell Optiplex 9010 AO computers with 3.1 GHz Intel Core i7 CPUs, 8 GB RAM, Microsoft Windows 10 Education 64-Bit (Version 10.0, Build 19,043). The stimuli were generated using MATLAB (R2015b, 2015) and the Psychophysics Toolbox extensions (Version 3.0.11; Brainard, 1997; Pelli, 1997). Screens were Dell Optiplex displays (resolution 1,920 × 1,080 pixels, 60-Hz refresh rate), headphones were Audio-Technica ATH-M20x headphones.

### Experiment 1: Stimuli and procedure

**Fig. 2:**
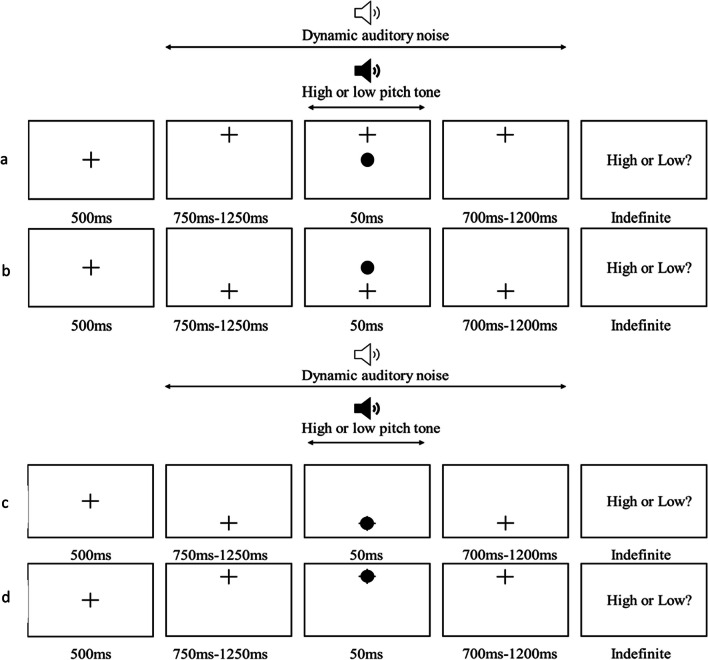
The panels depict examples of trials from the retinotopic and representational blocks of Experiment 1. From top to bottom: 2.a is a retinotopic „low‟ trial, 2.b is a retinotopic „high‟ trial, 2.c is a representational „low‟ trial, and 2.d is a representational „high‟ trial

In Experiment 1, participants were seated in front of the computer with their heads approximately 80 cm away from the screen and wearing the headphones. For the first 500 ms, they were presented with a grey screen with a black fixation cross in the centre, and were asked to keep their gaze on the cross for the duration of the trial. After 500 ms, the fixation cross was redrawn 5.2 visual degrees above or below the original fixation position. In the instructions, participants were asked to follow the cross to the new position with their eyes. Once the cross moved, there was a variable delay of 750 ms, 916 ms, 1,083 ms or 1,250 ms. During this delay, white noise was presented through the participants’ headphones. At the end of the delay, the audiovisual stimulus was presented. The visual component consisted of a black dot, 2 visual degrees in diameter. The auditory component was a pure sinusoidal tone that was either low or high in pitch (750 Hz or 1,500 Hz). Both the auditory tone and the visual target were presented concurrently for 50 ms, then offset. The white noise persisted before, during and after the tone for a total of 2,000 ms to mask the auditory stimulus and reduce participant accuracy to enable their discrimination task. In the representational block, the visual target was presented over the redrawn fixation position. This resulted in a visual target which was retinotopically central regardless of the position the participant had been instructed to fixate on but varied in representational screen elevation from trial to trial. In the retinotopic block, alternatively, the visual target was presented over the original fixation position on every trial. This resulted in a visual target which was always central on the screen (and so was constant in representational elevation) but varied in its retinotopic elevation from trial to trial. After being presented with the audiovisual stimulus, participants were presented with a two-alternative forced-choice (2AFC) task on the pitch of the tone and made responses using the arrow keys with their right hand. Example trials from the retinotopic and representational blocks of Experiment 1 can be seen in Fig. 2.

### Experiment 2: Stimuli and procedure

**Fig. 3:**
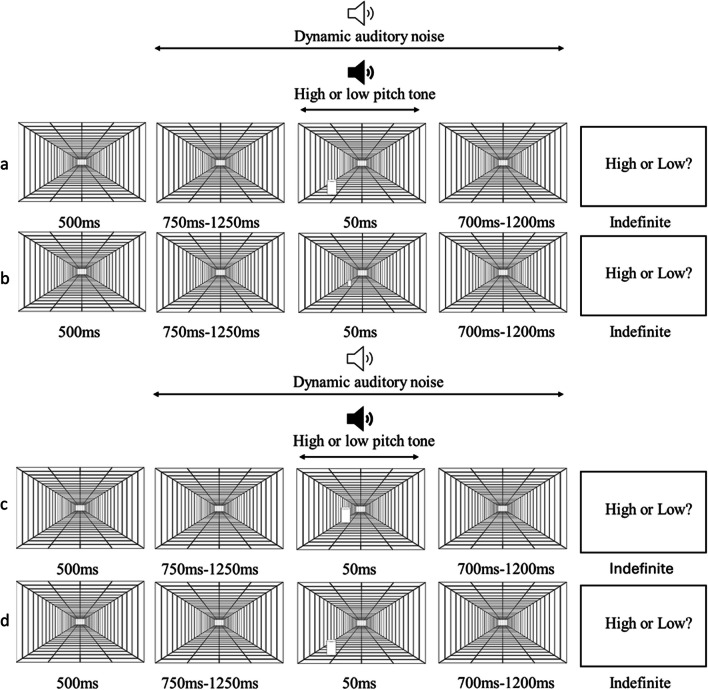
Panels depict examples of trials from the retinotopic and representational blocks of Experiment 2. From top to bottom: 3.a is a retinotopic big trial, 3.b is a retinotopic small trial, 3.c is a representational big trial, and 3.d is a representational small trial

In Experiment 2, participants had the same seating arrangement as in Experiment 1 and were instructed to keep their eyes on the central fixation cross on every trial. After viewing the same 500 ms of a grey screen with a central black fixation, participants were presented with a visual depth illusion. The illusion uses the convergence of parallel lines to give the impression to depth and was presented centrally. Objects placed into the illusion appear farther from the viewer the closer they are to the centre. The illusion persisted for a variable jitter of 750 ms, 916 ms, 1,083 ms or 1,250 ms. In addition to this, white noise was played while the illusion was present on screen. After the delay, the audiovisual stimulus was presented. The visual stimulus consisted of a 3D white cylinder object, presented either close to the edge of the visual depth illusion to appear nearer to the participant or close to the centre, producing the illusion that it was further away from the participant. The near cylinder was always 2.8 visual degrees in width and 4.2 visual degrees in height. The qualities of the far cylinder varied between the retinotopic and representational blocks. In the representational block, where the purpose was to hold retinotopic size constant, the far cylinder was the same visual degrees as the near. In this case, following Emmert’s Law, the far cylinder produced a representationally larger cylinder while the near cylinder produced a representationally smaller cylinder. In the retinotopic block, alternatively, the far cylinder was 1.27 visual degrees width and 1.66 visual degrees in height. The reduction in visual degrees for the far stimulus was consistent with the cylinder being moved further into depth, resulting in a change in retinotopic size without a corresponding change in the cylinder’s representational size. The visual stimulus was presented from all four corners of the illusion, in a randomised order. As in the above experiment, the auditory stimulus was a pure tone either low or high in pitch (750 Hz or 1,500 Hz). The audiovisual stimuli persisted for 50 ms and then disappeared. The white noise and depth illusion remained present until 2,000 ms had elapsed from the beginning of the trial. Participants then performed the same 2AFC task on the pitch of the auditory stimulus. An example of the big and small visual scene, for both retinotopic and representational size, can be seen in Fig. 3.

## Results

As described briefly in the design, the variables of pitch and the visual feature (elevation in Experiment 1, size in Experiment 2) were collapsed along congruent/incongruent lines according to the elevation/pitch and size/pitch CMCs to categorize trials as either congruent or incongruent. In elevation/pitch, congruent trials were high elevation/high pitch and low elevation/low pitch. In size/pitch, congruent trials were small size/high pitch and large size/low pitch. Mismatches between the visual and auditory feature were categorized as incongruent. Participant scores were measured in percentage of correct discriminations of pitch (accuracy). Comparisons were made using two-tailed paired t-tests.

### Planned comparisons of congruency effects

**Fig. 4:**
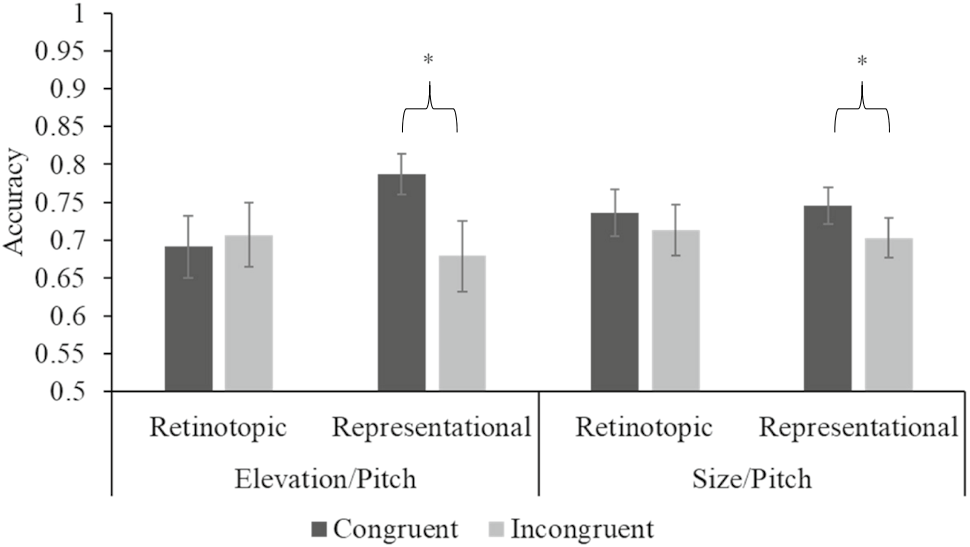
Fig. 4 Accuracy in judgements of the auditory feature in Experiments 1 and 2, split by congruency and feature type. Asterisks indicate significance of congruent vs. incongruent t-tests: *p < .05. Error bars are standard error

A paired two-tailed t-test comparing accuracy in congruent trials against incongruent trials within the representational block of Experiment 1 revealed a significant accuracy benefit for congruent trials (M = 0.787, SD = 0.139) over incongruent trials (M = 0.679, SD = 0.239), *t*(26) = 2.45, p = 0.021, Cohen’s d = 0.472. A paired two-tailed t-test comparing accuracy in congruent trials against incongruent trials within the retinotopic block of Experiment 1 revealed no significant difference in accuracy between congruent trials (M = 0.691, SD = 0.212) and incongruent trials (M = 0.707, SD = 0.219), *t*(26) = -0.364, p = 0.718. A paired two-tailed t-test comparing accuracy in congruent trials against incongruent trials within the representational block of Experiment 2 revealed a significant accuracy benefit for congruent (M = 0.746, SD = 0.123) trials over incongruent trials (M = 0.703, SD = 0.134), *t*(25) = 2.259, p = 0.032, Cohen’s d = 0.443. A paired two-tailed t-test comparing accuracy in congruent trials against incongruent trials within the retinotopic block of Experiment 2 revealed no significant difference in accuracy between congruent trials (M = 0.736, SD = 0.16) and incongruent trials (M = 0.713, SD = 0.172), *t*(25) = 1.603, p = 0.121. Figure 4 shows the results of the four paired comparisons.

## Discussion

We observed that crossmodal congruency effects manifest *only* when the visual features of elevation and size were present in representational features derived from environmental cues. This strongly suggests that the elevation/pitch and size/pitch crossmodal correspondence depends on real-world features, which environmentally constructed mentally represented features more closely reflect. Conversely, changes in the retinotopic features of elevation and size are not associated with any crossmodal interactions with pitch if the representational feature of the real-world aspect is held constant. Expanding this to the question of whether elevation/pitch and size/pitch rely more on environmental information or the retinotopic experience, it seems that these crossmodal correspondences are sensitive to the environmental features of the pairing, and the immediate retinotopic experience is less important, as long as the sensory experience induces a mental representation of the environmental feature which allows for congruency effects.

These results are also interesting compared with Carnevale and Harris (2016), who found that elevation/pitch was biasing judgements of spatial motion in both body-centric and gravity-centric reference frames. In contrast, we only find elevation/pitch CMC effects in the representational condition. We believe the most likely reason for this discrepancy is the way each study operationalized the conditions. As mentioned in the *Introduction*, Carnevale and Harris (2016) tilted participants to operationalize their design while our study does not. The use of tilt, and by extension, introduction of vestibular and proprioceptive feedback, may be causing this difference. Body representation utilizes a wide range of brain regions and produces an even wider number of effects, all of which may influence the elevation/pitch CMC (see Longo et al., 2010, for a detailed review of body representation).

We propose that this finding provides support for an environmental statistics account of crossmodal correspondence in these pairings. The environmental statistics hypothesis suggests that crossmodal correspondences are drawn from the environmental correlation of two features from different modalities. By repeatedly observing these correlations, an individual will internalize them as internal associations, to be observed in crossmodal congruency effects. If this was the case, crossmodal correspondences would likely be specific to the environmental correlation from which they were drawn. These environmental correlations are explained by physical laws such as larger objects resonating at lower pitches, or elevated objects being lighter, smaller, and resonating at higher pitches. As discussed earlier, retinotopic features do not necessarily reflect the veridical feature, and by extension the physical law. An object can be brought nearer to the eye to increase its retinotopic feature of size, but this would not alter the pitch of the object. We can say from this that the environmental hypothesis should only be observed in the representational construction of the veridical features. Our results are consistent with this explanation. Both experiments where the visual stimulus provided cues to induce a mental representation of the veridical or ‘real-world’ feature while the retinotopic experience was held constant produced crossmodal congruency effects.

We can also consider these results with regard to the structural hypothesis, which proposes that crossmodal correspondences are driven by neural encoding of the associated stimuli (Spence, 2011). While our results do not directly support or contradict a structural account of crossmodal correspondence in our CMC pairings, the finding that elevation/pitch and size/pitch follow environmental correlations suggests that any structural account is likely still environmentally driven. In other words, these crossmodal correspondences do not appear to be arbitrary. This is in opposition to many variations of the structural hypothesis which do argue for an essentially arbitrary correspondence such as theories suggesting that correspondence arises from a shared neural system for encoding magnitude (Smith & Sera, 1992; Walsh, 2003), or theories which attribute correspondence to the distance between brain regions which encode for each feature (Ramachandran & Hubbard, 2001). Our finding that elevation/pitch and size/pitch crossmodal correspondence appears to be specifically related to the feature as it exists in the environment suggests that any neural mechanism behind these crossmodal congruency effects is likely an adaptation to the environment rather than a coincidence. This is more in line with discussions by Parise (2016), who speculates on the possibility of a structural CMC account where they are innate but evolved in response to environmental correlations. Such a framework would also explain the variety of infant studies which show CMC effects at an age argued to be too early for environmental learning.

Finally, these results could be considered compatible with a semantic account of crossmodal correspondence for elevation/pitch and size/pitch, although they do not necessarily support it. There is strong evidence from studies of language that suggest linguistic labels are derived from representational features (Connell, 2019; Riordan & Jones, 2011; Sloutsky & Fisher, 2012). In other words, a person will never use the label ‘big’ to describe an object which occupies a large area retinotopically. Rather, these terms are used to describe the representational quality, likely because this representational quality is what is shared by other observers, and so is typically more meaningful in communication. On the other hand, the presence of these CMC associations in non-human animals remains a problem for a semantic account of the elevation/pitch and size/pitch CMCs and may alternatively suggest that the semantic hypothesis is incorrectly attributing these CMCs to shared linguistic labels, when these shared linguistic labels are actually derived from a cognitive process shared by many animals (Korzeniowska et al., 2019, 2022).

These results should be treated cautiously, however, when it comes to expanding these interpretations beyond correspondences involving pitch. Pitch is one of the most studied features in CMCs, possibly because of how reliably it produces CMC effects. Moreover, elevation/pitch and size/pitch are some of the likeliest examples of environmental correlations (Deroy et al., 2018; Spence, 2020b). Elevation/pitch has been demonstrated in the environment by Parise et al. (2014), and size/pitch likely requires no empirical proof, since it relies on the physical characteristics of sounding bodies. It is possible that pitch-based correspondences are different from other CMCs due to their highly reliable environmental correlation, and so rely more on environmental information, in line with our findings that the representational feature of the stimulus is what creates the congruency effect. Future research in this line of inquiry would do well to examine CMC pairings with less intuitive environmental correlations such as brightness/loudness.

In addition to considering the phenomenological origins of elevation/pitch and size/pitch, we can consider the timeline of information availability in the sensory system to speculate on the neural basis of this effect. As discussed briefly in the *Introduction*, veridical features of the environment are not immediately available (and arguably never become available) to a viewer. The viewer must construct a mental representation of the veridical features through a combination of sensory information (Kanai & Verstraten, 2006; Perdreau & Cavanagh, 2013; Vanston et al., 2023). For elevation, this could be a combination of retinal position, vestibular feedback, and contextual cues like the position of the ground or uprightness of objects. Similarly, mental representation of size is a combination of retinal size, stereopsis, monocular depth cues, and again contextual cues like knowledge of the object’s typical qualities such as how it fits into the hand. This construction of representational information takes time, and so the representational feature will not be available for multisensory integration until it is constructed. We can use our knowledge of which features are used in crossmodal correspondence to make arguments for a minimum speed of elevation/pitch and size/pitch CMC congruency effects, based on when this information becomes available.

First, we can consider the timeline of representational elevation in the brain. Interestingly, despite elevation being seemingly ‘simpler’ than size in neural complexity, the question of how the brain maintains a spatiotopic map of the world (including concepts like ‘up’) from a retinotopic representation in V1 which changes with each saccade is still heavily debated. Important work with non-human apes suggests that there are so-called ‘real position’ cells in some regions of the brain, which encode for spatial position independent of retinotopic changes. These cells are found throughout the brain but regions where this spatial position can be encoded by a single cell, the most likely regions for this process, include the intraparietal sulcus and parieto-occipital sulcus (Duhamel et al., 1997; Galletti et al., 1993). Although there is no time-course available for this specific process, we can speculate based on these regions that representational elevation is not available to the brain until 100–140 ms in V6 at the earliest (Pitzalis et al., 2013a, 2013b). In addition, V6 shows increased activity a second time to a visual stimulus approximately 100 ms after initial activation at 100–140 ms. Elevation/pitch would require this 100–140 ms at a minimum to begin integrating representational elevation with pitch.

Second, we can look at the time-course for the calculation of representational size. Representational size is created through a process called size constancy, where the brain maintains a concept of an object’s size despite retinotopic changes. Although there is good evidence that V1 contributes directly to size constancy processing (Novilleo et al., 2024; Sperandio et al., 2012), other studies suggest that size constancy in V1 is under the top-down control of higher regions (Chen et al., 2019; Qian & Yazdanbakhsh, 2015; Tanaka & Fujita, 2015). Chen et al. (2019) used EEG to examine the time-course of size constancy in V1 and suggested that it takes at least 150 ms to process representational size. We can speculate from this that size-/pitch-related crossmodal congruency effects should take at least 150 ms to begin.

From these timelines, we can discuss how our work integrates with current evidence for low-level early crossmodal correspondence effects. Recent studies of low-level crossmodal correspondence (Kovic et al., 2010; McEwan et al., 2024; Sciortino & Kayser, 2023; Zeljko et al., 2019) suggest that crossmodal correspondence effects occur at the level of the primary sensory cortices or earlier. Although our work is largely consistent with this possibility, it does not necessarily support it. It does qualify it, however, in suggesting that even if the elevation/pitch and size/pitch crossmodal correspondences occur at the level of the primary sensory cortices, the manifestation of the effect is under some form of top-down control. Part of this top-down control is the requirement for the formation of a representational feature before multisensory integration.

There are some limitations with the current design which require acknowledgement, however. We did not verify that participants completed the eye movements of the elevation/pitch task as instructed, and so it is possible that they did not make the required gaze shifts to operationalize the retinotopic/representational conditions. We believe that this is unlikely, because the redrawing of the fixation cross would induce an exogenous shift of attention and accompanying eye movement towards the new fixation position. Participants would have to actively resist shifting their gaze as cued by the task. Related to the question of gaze shifts is the question of inhibition of return (IOR), an inhibition of processing in a region of space which has previously been attended. It is possible that the paradigm requiring participants to shift their gaze away from the centre of the screen to the edge, then respond to an audiovisual target, the visual component of which was presented in the centre of the screen, may have induced IOR in the visual component. The degree to which this would have influenced the results is questionable, however. Many behavioural studies such as Helbig and Ernst (2008), Parise and Spence (2012), Evans and Treisman (2010), and Zeljko et al. (2019), as well as neuroimaging studies (Kovic et al., 2010; Sciortino & Kayser, 2023), argue that these crossmodal correspondence effects are pre-attentive, and so in our study they should not be influenced by IOR.

It is also worth noting that the size/pitch experiment was conducted in pictorial space (the 3D impression from a 2D picture) rather than real space, and thus not processed identically to real space. Critically, individuals do not necessarily resolve ambiguities when interpreting pictorial scenes in the same way. Although they will still do so within veridical boundaries, there is a range of possible perspectives and distances which can produce the same pictorial 2D image, and so they will show variation in their interpretation, something referred to as ‘the beholder’s share’. Koenderink et al. (2001) for instance, suggest that people may take up a mental perspective within a pictorial image, which they can then adjust to make sense of the available depth cues. Although this does not undermine the findings of the current study, we cannot definitively say whether these results would extend to an experiment using stereoscopic depth, either in the real world or simulated through a stereoscope/VR system.

Overall, our results provide tentative support for an environmental hypothesis of crossmodal correspondence in elevation/pitch and size/pitch, and clarify possible interpretations of the structural hypothesis. The absence of crossmodal congruency effects when mental representations of the feature was held constant suggests that the CMC congruency effects of these pairings rely on top-down interpretation of the scene to determine the value of the feature pairings. Additionally, these findings provide suggestions for the temporal profile of elevation/pitch and size/pitch crossmodals correspondence through interpretation of the required features.

## Data Availability

All experimental data has been made available via the Open Science Framework at 10.17605/OSF.IO/EGXYZ.

## Declarations

### Open practices statement

All experimental code and data have been made available on the Open Science Framework at .DOI 10.17605/OSF.IO/EGXYZ

No experiments or analyses were preregistered.
